# Sexual Dimorphism on a Conserved Scaffold: Insights from the Floral Ontogeny of *Eurychorda* (Restionaceae: Poales)

**DOI:** 10.3390/plants15010097

**Published:** 2025-12-28

**Authors:** Constantin I. Fomichev, Barbara G. Briggs, Dmitry D. Sokoloff

**Affiliations:** 1 Department of Higher Plants, Faculty of Biology, Lomonosov Moscow State University, Moscow 119234, Russia; 2 National Herbarium of New South Wales, Botanic Gardens of Sydney, Australian Botanic Garden, Mount Annan, NSW 2567, Australia; barbarab@smartchat.net.au; 3 School of Plant Sciences and Food Security, Faculty of Life Sciences, Tel Aviv University, Tel Aviv 6997801, Israel; dsokoloff@tauex.tau.ac.il

**Keywords:** Australia, evolutionary parallelism, flower, inflorescence, modular segmentation, pistillode, pollen release, spikelet, staminode, wind pollination

## Abstract

Angiosperms include many taxa with dimorphic unisexual reproductive structures. These are well studied in some grasses, with maize as a key model, but other wind-pollinated lineages in Poales remain less explored. Within Poales, the family Restionaceae has the highest known proportion of dioecious species. In its Australian subfamily Leptocarpoideae, the sexually dimorphic *Leptocarpus denmarkicus* has raised questions about the basic flowering unit and the developmental basis of dimorphism. Here, we analyze inflorescence architecture and floral development in *Eurychorda complanata*, the sister lineage to the remainder of Leptocarpoideae. Using comparative morphology, light microscopy and scanning electron microscopy, we reconstruct synflorescence topology, floral organography, and ontogeny in both sexes and compare them with those in *L. denmarkicus*. In *Eurychorda*, both sexes produce polytelic paniculate synflorescences with distinct inhibition zones and many-flowered simple spikelets as the basic flowering unit. Male and female spikelets bear up to 50 and up to 15 fertile flowers, respectively. Male flowers have two stamens and a dimerous pistillode, whereas female flowers possess two long filamentous staminodes and a dimerous gynoecium. Ontogenetic series show that flowers of both sexes initiate both androecial and gynoecial structures, and that functional unisexuality is achieved through late arrest of the organs of one sex. Defining spikelets as racemose axes with lateral sessile flowers clarifies homologies of reproductive structures and supports reinterpretation of the dimorphic female unit in *L. denmarkicus* as a derived compound spike generated through shifts in branching rank and the timing of lateral initiation. The compound female spike of *L. denmarkicus* has a striking overall similarity to the simple female spikelet in *Eurychorda*, illustrating fascinating parallelism in the evolution of reproductive organs within Restionaceae and Poales more broadly. At the male side, *Eurychorda* achieves anther exsertion via filament elongation, whereas in *L. denmarkicus* filaments are very short and anthers remain within the perianth, but male spikelets sit on long, flexible peduncles that invert the spikelet and promote trembling, thereby ventilating the perianth chamber and aiding pollen escape. These two solutions—filament elongation versus spikelet-peduncle flexibility—represent alternative strategies of pollen release in wind-pollinated flowers.

## 1. Introduction

Unisexual flowers and dimorphic unisexual inflorescences are recurrent themes across angiosperms [1] and are best studied in grasses, with monoecious maize as a canonical model. In maize, male (tassel) and female (ear) flowers arise on the same individual through selective suppression of opposite-sex organs [2], coordinated by genetic and hormonal pathways, including *tassel seed* genes, brassinosteroid- and jasmonic acid-related signaling [3,4,5,6]. This framework shows how unisexuality and sexual dimorphism can evolve and diversify in wind-pollinated plants. However, maize and most other grass models are monoecious, and dioecy is comparatively rare in Poaceae. By contrast, unisexual flowers and dioecy are widespread in Restionaceae, a related family within Poales, making them an excellent system for studying the evolution of sexual dimorphism in strictly dioecious wind-pollinated plants.

Within Poales, which includes grasses, sedges, and other ecologically dominant lineages [7], Restionaceae are predominantly dioecious, rush-like, wind-pollinated plants with typically small flowers [8]. The family has two Southern Hemisphere diversity centers: Australia, rich in genera, and South Africa’s Cape region, rich in species [9]. Despite substantial descriptive and taxonomic work [8,10,11,12,13], the evolutionary significance of inflorescence architecture in Restionaceae remains underexplored. A central problem is the homology and hierarchical rank of the spikelet: is the unit recognized in taxonomical research a simple racemose axis bearing lateral, sessile flowers (a simple spikelet), or a compound structure assembled from reduced one-flowered modules? Recent work on *Leptocarpus denmarkicus* sharpened this issue by showing pronounced sexual dimorphism: males bear simple spikelets, whereas females carry compound spikes comprising reduced single-flower spikelets [13]. This finding raises broader questions about developmental routes to dimorphism and diversification of reproductive shoots across the family.

**Figure 1:**
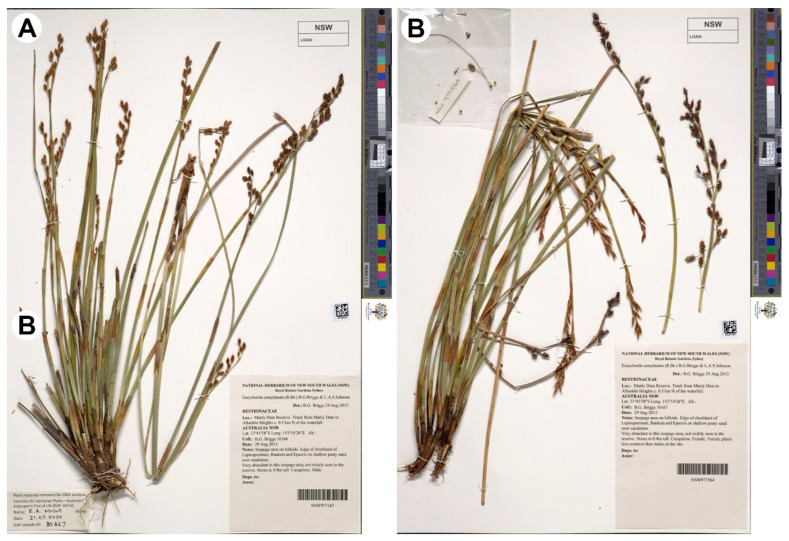
Habit of *Eurychorda complanata*. (**A**) Voucher specimen of the examined male plant (NSW 977167). (**B**) Voucher specimen of the examined female plant (NSW 977564).

Within Leptocarpoideae, the monotypic eastern Australian genus *Eurychorda* B.G.Briggs & L.A.S.Johnson (Figure 1) is recovered as sister to the remainder of the subfamily in plastid-based phylogenies with broad Australian sampling [14]. *Eurychorda complanata* (R.Br.) B.G.Briggs & L.A.S.Johnson is readily recognized by flattened culms with pillar cells together with dimerous flowers [8]. Several reproductive characters of *Eurychorda* have been discussed as potentially ancestral for the family [14]. However, its inflorescence organization and floral development have not been documented in detail, limiting assessments of character polarity and the origins of sexual dimorphism in Leptocarpoideae. Critically, sister-group position does not by itself imply plesiomorphy of all character states: extant early-branching lineages are not “primitive,” and ancestral states require explicit character optimization rather than tree position alone—the so-called primitive lineage fallacy [15]. Analogous cautions are relevant to discussions on *Amborella* and early angiosperm evolution [16].

Here we integrate morphological, anatomical and ontogenetic data to analyze male and female inflorescence architecture, floral structure and development sequences in *E. complanata* and compare these features with those of *L. denmarkicus* [13]. We (i) adopt a strict spikelet definition appropriate for Restionaceae, (ii) test alternative interpretations of the female flowering unit (simple spikelet versus compound spike), and (iii) identify developmental pathways consistent with sexual dimorphism, including how wind-pollination mechanics interface with these architectures. Framing the problem in this way enables an initial polarization of character transformations within Leptocarpoideae using the position of *Eurychorda* [14], while clarifying homology and terminology that have historically obscured comparison across Poales.

## 2. Results

### 2.1. Inflorescence Morphology

**Figure 2:**
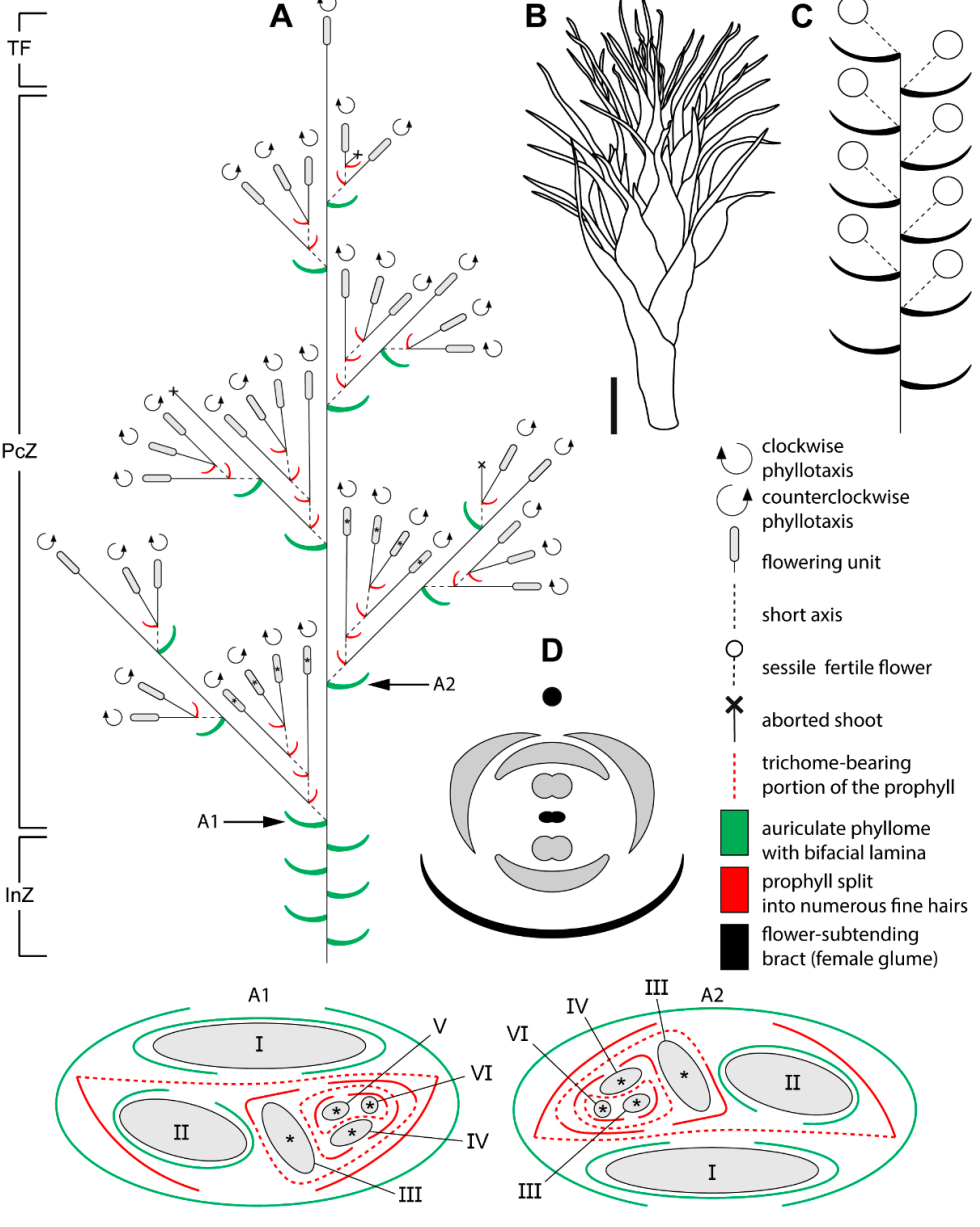
Schematic representation of the male synflorescence in *Eurychorda complanata*. (**A**) Overall synflorescence structure. Arrows indicate inflorescence modules illustrated in A1 and A2, with asterisks marking flowering units corresponding to those shown in A1 and A2. Roman numerals indicate the branching order. (**B**) Habit of a male flowering unit. (**C**) Diagram of the male flowering unit. Note that the actual number of flowers is much higher than illustrated here. (**D**) Diagram of the male flower. The black circle represents the spikelet axis, and the black phyllome denotes the flower-subtending bract (male glume). InZ, inhibition zone; PcZ, paraclade zone; TF, terminal flowering unit. Scale bar: 1 mm (**B**).

**Figure 3:**
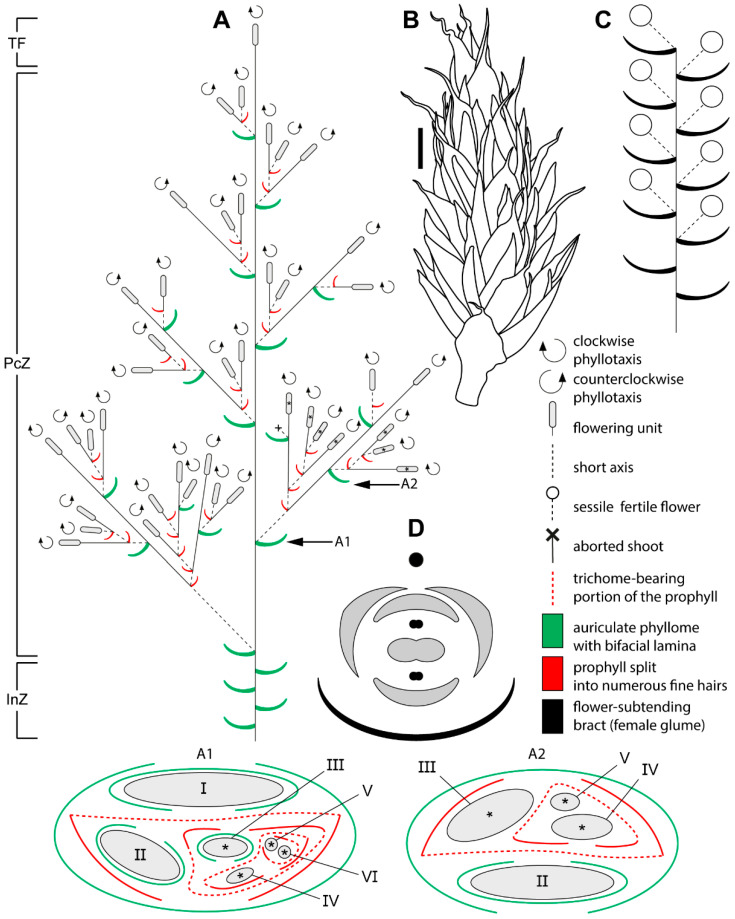
Schematic representation of the female synflorescence in *Eurychorda complanata*. (**A**) Overall synflorescence structure. Arrows indicate inflorescence modules illustrated in A1 and A2, with asterisks marking flowering units corresponding to those shown in the A1 and A2. Roman numerals indicate the branching order. (**B**) Habit of a female flowering unit. (**C**) Diagram of the female flowering unit. Note that the actual number of flowers is much higher than illustrated here. (**D**) Diagram of the female flower. The black circle represents the spikelet axis, and the black phyllome denotes the flower-subtending bract (female glume). InZ, inhibition zone; PcZ, paraclade zone; TF, terminal flowering unit. Scale bar: 1 mm (**B**).

**Figure 4:**
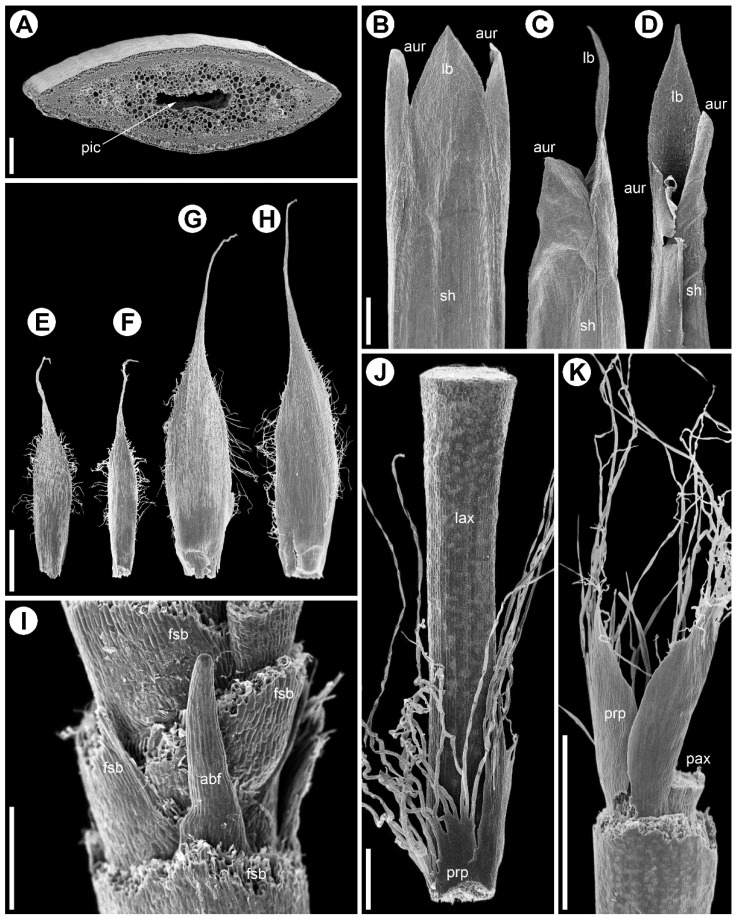
Stem and leaf morphology in *Eurychorda complanata* (SEM). Female (**A**–**D**,**G**–**K**) and male (**E**,**F**) plants; (**A**) internode cross-section; (**B**–**D**) vegetative leaves from the inhibition zone (similar leaves occur along the synflorescence main axis up to the apex and at basal paraclade nodes, except at prophyll-bearing nodes). (**B**) Abaxial view, (**C**) lateral view, and (**D**) adaxial view. (**E**–**H**) Flower-subtending bracts of male (**E**,**F**) and female (**G**,**H**) flowers. (**E**,**G**) Abaxial side, (**F**,**H**) adaxial side. (**I**) Base of a female spikelet with partly removed bracts showing an aborted flower. (**J**,**K**) Prophyll split into numerous fine hairs, viewed from the parent-axis side (**J**) and away from it (**H**). abf, aborted flower; aur, auricle; fsb, flower-subtending bract; lb, leaf blade; lax, lateral axis; pax, parent axis; pic, pith cavity; prp, prophyll; sh, sheath. Scale bars; 200 µm (**A**); 1 mm (**B**–**H**,**K**), 300 µm (**I**,**J**).

Male and female reproductive shoots are similar. Plants are caespitose, forming large, dense tussocks. From the subterranean rhizome arise assimilating aerial shoots, each terminating in a polytelic synflorescence (Figure 1, Figure 2A and Figure 3A) whose main axis ends in a spikelet (the flowering unit). The lower portions of the aerial shoots have short internodes. Stems are flattened and contain a central cavity (Figure 4A). Leaves are appressed to the culm and consist of a sheath with a very short, closed base bearing a pair of auricles that flank the blade (Figure 4B–D). The blade is bifacial and deltoid, and a ligule is absent. No branching occurs in the axils of vegetative leaves of aerial shoots, defining the inhibition zone of the synflorescence (Figure 2A and Figure 3A).

Flowers are grouped into many-flowered spikelets (Figure 2B,C and Figure 3B,C) and are borne in the axils of oblong bracts, termed *glumes* in the Restionaceae literature [8]. Each bract has a long blade expressed as a narrow cylindrical outgrowth approximately three quarters of the bract length; a row of trichomes develops along the bract margin (Figure 4E–H). Within spikelets, bracts are arranged spirally and the internodes are short.

**Figure 5:**
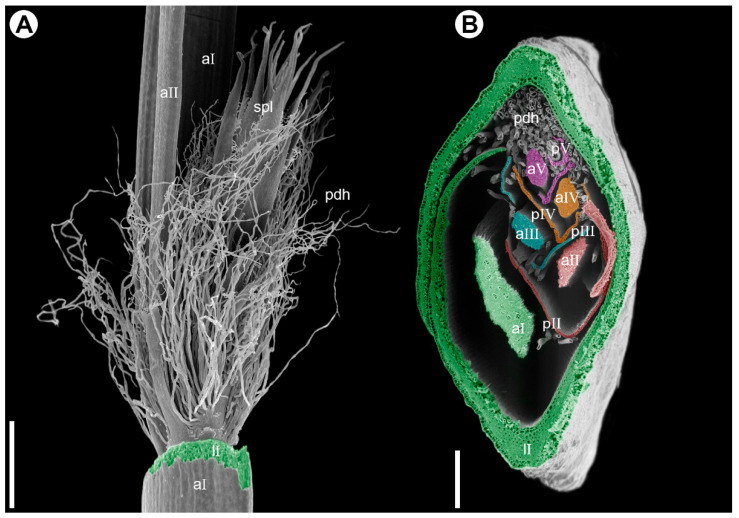
Branching pattern in synflorescence of *Eurychorda complanata* (SEM). (**A**) Portion of the male synflorescence main axis with its leaf removed to show the base of the paraclade. The first-order paraclade terminates in a spikelet (not shown) and bears a second-order paraclade, which also terminates in a spikelet. Hypopodium (the portion of stem below the prophyll) is short in all paraclades, and the middle portion of the prophyll is split into numerous fine hairs. (**B**) Cross-section of the female synflorescence main axis showing the branching pattern. Axes and their leaves are artificially colored: first order, green; second order, red; third order, turquoise; fourth order, orange; fifth order, magenta. aI–aV, axes of subsequent branching orders; lI, leaf on the first-order axis; pII–pV, prophylls of subsequent lateral axes; pdh, prophyll-derived hairs; spl, spikelet. Scale bars: 1 mm (**A**), 300 µm (**B**).

Bracteoles are absent. The axils of several lower bracts contain abortive flowers (Figure 4I). Male spikelets bear up to 50 fertile flowers, whereas female spikelets bear up to 15. Spikelets are also borne on lateral axes (paraclades), which form a paraclade zone below the terminal flowering unit and then abruptly transition basipetally into an inhibition zone (Figure 2A and Figure 3A). Paraclades may be branched to the fifth order in both male and female synflorescences. The degree of branching within the synflorescence increases from top to bottom, producing a panicle of spikelets (Figure 2A and Figure 3A). Each paraclade has a short hypopodium followed by long internodes. Branching occurs in the axils of both the prophyll and the subsequent leaves. Because hypopodia are short and branching can occur in the prophyll axil, several lateral axes may appear to arise from a single leaf axil of the main axis (Figure 5). The lateral axes emerge either to the left or to the right of the prophyll in branches of successive orders thus forming a zigzag pattern (Figure 5B).

Phyllotaxis is distichous along the synflorescence axes and transitions to a spiral arrangement in flowering units (Figure 2(A1,A2) and Figure 3(A1,A2)). The ontogenetic spiral of the flowering units may be clockwise or anticlockwise, with antidromous orientation between axes of successive orders (Figure 2A and Figure 3A). Several leaf types are present within the synflorescence (Figure 4). The main axis bears leaves similar to those in the inhibition zone (Figure 4B–D). In prophylls, the two margins are hyaline and membranous, whereas the middle portion is split into a dense fringe of long hairs (Figure 4J,K). Because hypopodia are short and branching is prophyllate, prophylls of successive lateral axes form an intricate complex of numerous hairs (Figure 5A), complicating analysis of synflorescence architecture.

### 2.2. Floral Organography

**Figure 6:**
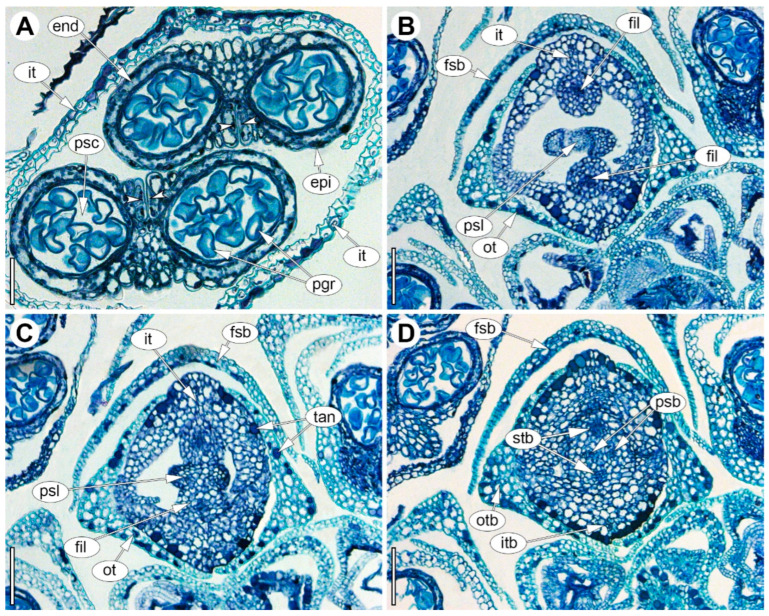
Anatomy of the male flower of *Eurychorda complanata* in transverse sections arranged from apex to base. (**A**) Section of the androecium at a level above the connective, showing two bisporangiate anthers. (**B**) Basal part of the androecium with two stamen filaments opposite the inner tepals and a dimerous pistillode at the flower center. (**C**) Separation of the androecium with two stamen filaments from the pistillode. (**D**) Upper part of the receptacle; delimitation of the inner tepals. end, endothecium; epi, epidermis; fil, filament; fsb, flower-subtending bract; it, inner tepal; itb, inner tepal bundle; ot, outer tepal; otb, outer tepal bundle; pgr, pollen grain; psb, pistillode bundle; psc, pollen sac; psl, pistillode; stb, stamen bundle; tan, tannins. Arrowheads indicate the longitudinal slit of anther dehiscence. Scale bars: 50 µm (**A**), 100 µm (**B**–**D**).

Male flowers are almost dissymmetrical, dimerous, and tetracyclic (Figure 2D and Figure 6); the perianth comprises two whorls with tepals free at the base so that the outer pair occupies transverse positions while the inner pair lies in the median plane, the adaxial inner tepal consistently overlapping the abaxial one (Figure 6B,C). In transverse sections, the outer tepals are V-folded (conduplicate) and keeled, whereas the inner tepals are not longitudinally folded (Figure 6B). The perianth opens at anthesis. Correspondingly, two stamens are situated in the median plane opposite the inner tepals (Figure 6A,B); each stamen is differentiated into filament and anther, and the anthers are bisporangiate, forming a single theca that dehisces by a longitudinal, introrse slit directed toward the center of the flower (Figure 6A). The anther is attached to the filament approximately at the mid-length or slightly shifted toward the base, while the filaments remain entirely free. Consistent with this, the anther wall shows flattened epidermal cells overall, but larger, irregular cells adjacent to the dehiscence slit, along which the epidermal cells bear numerous small ridges; the endothecium comprises large, rectangular cells in cross-section, the middle layer is obliterated, and no tapetum was detected in the available material (Figure 6A). The gynoecium of the male flower is reduced to a pistillode formed by two fused, reduced carpels (Figure 6B,C). Tannins are present in the epidermis of outer and inner tepals, the pistillode, and the anther. Each tepal and stamen is supplied by a single bundle, while the pistillode receives two bundles along the radii of the reduced carpels (Figure 6D).

**Figure 7:**
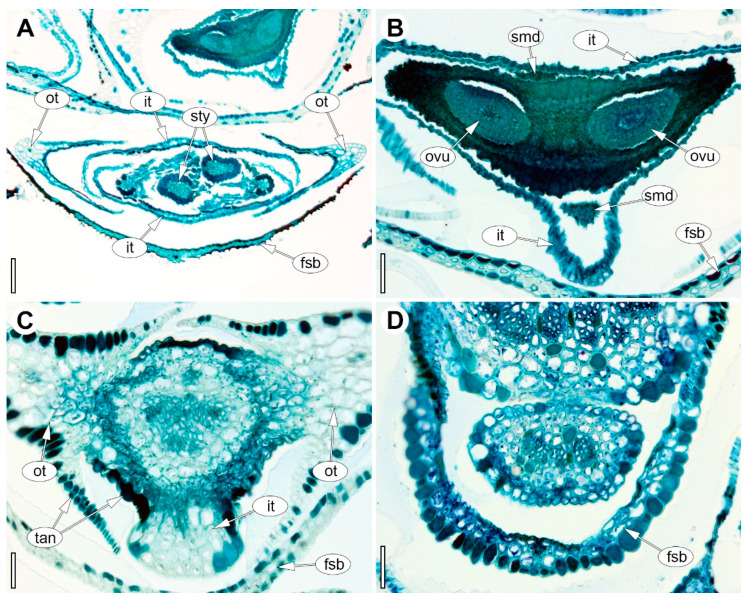
Anatomy of the female flower of *Eurychorda complanata* in transverse sections arranged from apex to base. (**A**) Asymplicate zone with two stylodia. (**B**) Synascidiate zone of the dimerous gynoecium. (**C**) Basal region of the receptacle showing the onset of delimitation of the two outer tepals. (**D**) Floral base prior to perianth delimitation. fsb, flower-subtending bract; it, inner tepal; ot, outer tepal; ovu, ovule; smd, staminode; sty, stylodium; tan, tannins. Scale bars: 100 µm (**A**), 50 µm (**B**–**D**).

Female flowers are dissymmetrical (slightly zygomorphic), dimerous, and tetracyclic (Figure 3D and Figure 7); again, the perianth comprises two whorls, with the outer tepals transverse and the inner tepals median (Figure 7A), and notably, the separation of the outer tepals from the receptacle begins at their margins (as seen in ascending series of cross sections). As in the male flower, the adaxial inner tepal overlaps the abaxial one in bud; likewise, the outer tepals are V-folded and keeled, the inner tepals are not longitudinally folded (Figure 7A). The inner perianth whorl does not open at anthesis. Instead of fertile stamens, the androecium bears two staminodes in the median radii (Figure 7B), each with a long filament and a minute anther. The gynoecium is syncarpous with a superior, bilocular ovary formed by two carpels located in the radii of the outer tepals (Figure 7B). The dorsal sides of the carpels are slightly shifted toward the adaxial side, which imparts slight zygomorphy (Figure 7B). A long asymplicate zone is expressed as two stigmatic branches (Figure 7A). Epidermal cells of both adaxial and abaxial tepal surfaces contain abundant tannins, rendering the protoplasts almost black (Figure 7A,C); additionally, the outer epidermis of the gynoecium is predominantly bilayered and locally multilayered (Figure 7B). Finally, the vascular architecture is consistent with the floral plan: each tepal and each staminode is supplied by a single bundle, whereas the gynoecium carries four bundles associated with ovule vascularization, plus two dorsal bundles that extend into the stylodia.

### 2.3. Flower Development

**Figure 8:**
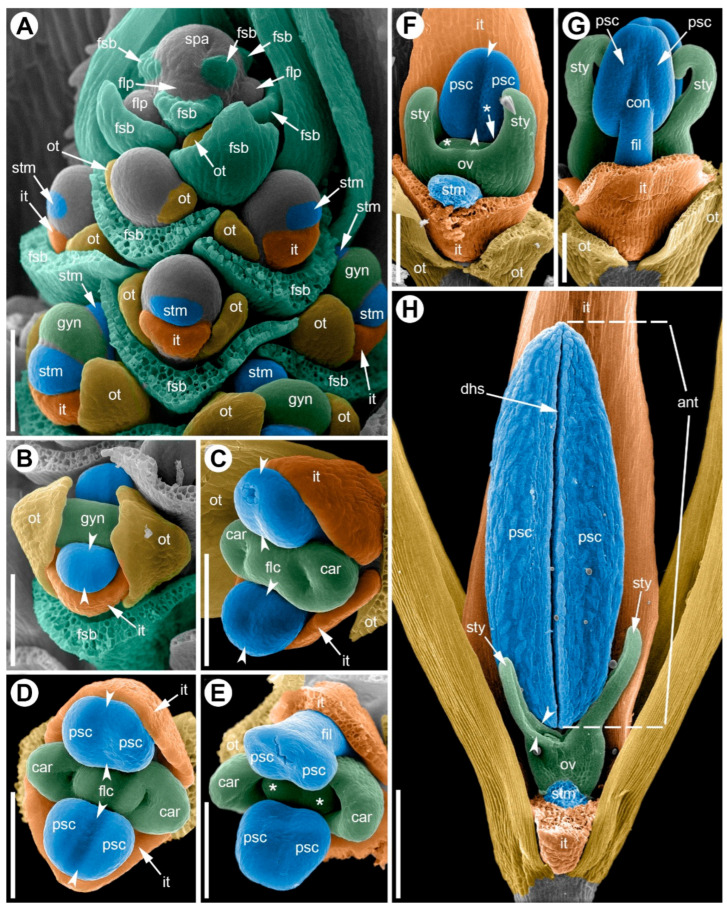
Development of the male flower of *Eurychorda complanata* (SEM). (**A**) Apex of a male spikelet with young flowers at successive developmental stages. (**B**) Top view of a flower at the stage of stamen initiation. (**C**) Early differentiation of the anther into two microsporangia; initiation of two carpels whose development will subsequently arrest. (**D**–**G**) Successive stages of longitudinal elongation of the anthers and filaments. (**H**) Mature (pre-anthetic) anther; gynoecium development arrested. One inner tepal and one stamen have been removed to reveal the pistillode. Arrowheads indicate the boundaries of the longitudinal furrow separating the pollen sacs. Asterisks mark young ovules that will soon become arrested in development. ant, anther; car, carpel; dhs, dehiscence slit; fil, filament; flc, floral center; flp, floral primordium; fsb, flower-subtending bract; gyn, gynoecium; it, inner tepal; ot, outer tepal; ov, ovary; psc, pollen sac; spa, spikelet apex; stm, stamen; sty, stylodium. Scale bars: 100 µm (**A**–**G**), 300 µm (**H**).

Male flower development begins with the floral primordium becoming isolated from the inflorescence apex by a narrow furrow while the subtending bract already encircles it (Figure 8A). As this happens, two outer-whorl tepals arise first as small, transversely arranged protrusions at the periphery of the floral apex (Figure 8A), and once these are fully delimited, the inner tepal on the abaxial side initiates (Figure 8A), followed shortly by the adaxial inner tepal.

The androecium arises as two primordia opposite the inner tepals (Figure 8A). At initiation, each stamen appears as a transversely elongated ridge whose length is about 1.5× its width. The two stamen primordia are separated by the developing gynoecium, which at this time forms a massive ridge (Figure 8B). The androecial primordia enlarge, and a longitudinal furrow develops on their inner side, dividing each anther into two equal halves (Figure 8C). During early androecial development, filament growth is weak and most expansion occurs in the anther (Figure 8C–G). Filaments then elongate rapidly approaching anthesis and release the developed anthers beyond the flower.

Two carpels are initiated (Figure 8C). The central portion of the gynoecium is occupied by the floral center (Figure 8C). The dorsal part of the carpels is separated from the floral center by a crescent-shaped furrow (Figure 8C,D). Carpel margins begin to elongate longitudinally, with growth especially pronounced on the dorsal side (Figure 8D–F), thereby forming the asymplicate zone composed of two stylodia (Figure 8G). In each locule, a single ovule initiates at the cross-zone (Figure 8E). Gynoecium development halts at this stage: the carpel margins do not fuse, and the ovary locules remain open (Figure 8H).

**Figure 9:**
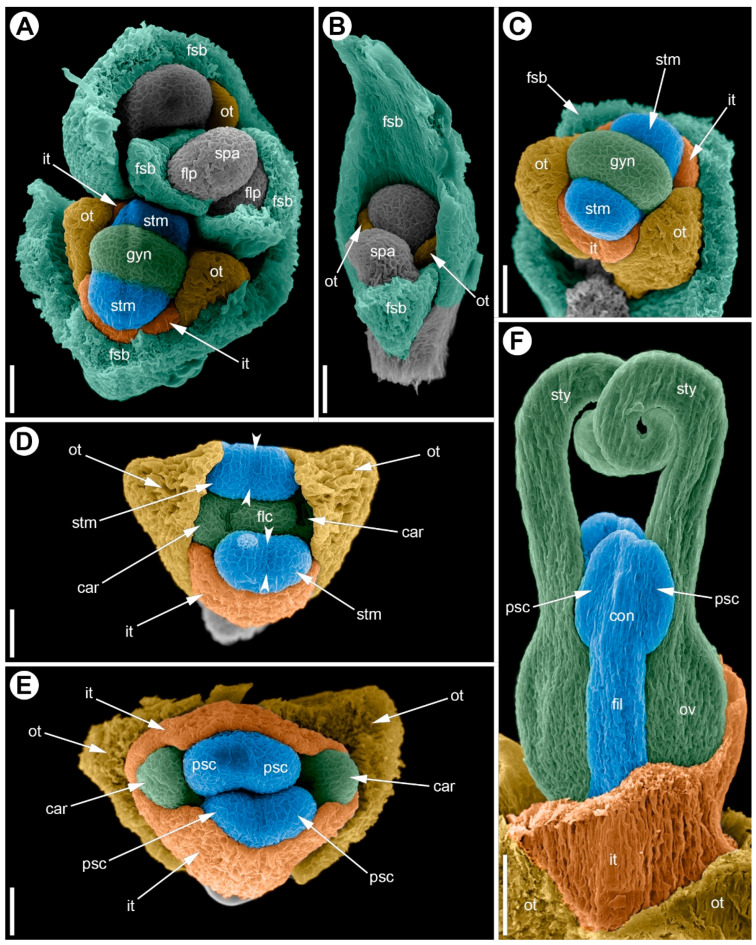
Development of the female flower of *Eurychorda complanata* (SEM). (**A**) Top view of the female spikelet showing the onset of floral primordium delimitation from the inflorescence apex. (**B**) Flower at the stage of delimitation of the two outer tepals. (**C**) Flower with the perianth delimited; staminodes are initiated opposite the inner tepals; the gynoecium is at early ridge stage. (**D**) Top view of a flower with two initiated carpels. (**E**) Top view of a female flower in which, at this stage, the staminodes still outgrow the carpels and meet above them, closing the floral center; the carpel tips remain visible. (**F**) Stages of longitudinal growth of the asymplicate zone of the gynoecium; carpel tips are spiral-coiled. Arrowheads indicate the boundaries of the longitudinal furrow separating the pollen sacs. car, carpel; con, connective; fil, filament; flp, floral primordium; fsb, flower-subtending bract; gyn, gynoecium; it, inner tepal; ot, outer tepal; ov, ovary; psc, pollen sac; spa, spikelet apex; stm, stamen; sty, stylodium. Scale bars: 30 µm (**A**–**E**), 100 µm (**F**).

Female flower development begins with the separation of a floral primordium in the axil of a bract (Figure 9A). The primordium arises on the periphery of the inflorescence apex as a small mound delimited by a transverse furrow (Figure 9A); by this time the bract already encircles the primordium. The first organs to initiate are the two outer tepals, which appear on the adaxial side of the flower as two small bumps (Figure 9B). Next, the inner tepals begin to differentiate; they arise in the median plane opposite each other (Figure 9C). By the time the androecium is initiated, the outer tepals start to grow radially along the midrib, forming keels (Figure 9C).

The androecium differentiates in the radii of the inner tepals as two primordia (Figure 9C). At inception, these appear as transversely elongated ridges (length roughly twice their width) separated by the gynoecium (Figure 9C). Soon, a longitudinal furrow becomes visible in the middle of each ridge, dividing it into two halves (Figure 9D). At early stages, the androecial primordia elongate longitudinally and arch over the developing gynoecium, meeting at the center with their anthers appressed to one another (Figure 9D,E). Subsequent growth shifts primarily to the filaments (Figure 9F), after which stamen development arrests, resulting in staminodes.

The gynoecium is initiated at the center of the flower, with carpels in the radii of the outer tepals, as a transversely elongated groove (Figure 9C). At early stages, the gynoecium is dumbbell-shaped in top view: the massive floral center occupies about one-third of the gynoecium and is flanked by the developing carpels. A narrow, crescent-shaped furrow then develops; its inner side remains part of the floral center and later bears the ovule, whereas its outer side is continuous with dorsal parts of the carpels (Figure 9D). The carpels enlarge, and enhanced longitudinal growth produces the asymplicate zone. Distal parts of the stylodia coil helically toward the floral center (Figure 9F). The two stylodia may develop evenly, but in some cases, one elongates faster than the other.

## 3. Discussion

**Inflorescence architecture and dimorphism.** *Eurychorda* and *Leptocarpus denmarkicus* share a conserved architectural template: polytelic, paniculate synflorescences are preceded by a distinct inhibition zone; paraclades possess short hypopodia; and branching takes place in the prophyll axil as well as in the axils of subsequent leaves in paraclades and the leaves along the main inflorescence axis. This pattern fits the general concept of polytelic synflorescences with an inhibition zone and hierarchically organized paraclade zone as elaborated in inflorescence morphology [17,18,19,20]. Comparable synflorescence architecture, including an inhibition zone and prophyll-based branching, has been documented in other poalean lineages [18,21] and specifically in Restionaceae [11,12,13]. Against this common background, the structure of flowering units differs between taxa and sexes. In *L. denmarkicus*, male plants produce true multi-flower spikelets, whereas the female unit is a compound spike assembled from reduced, single-flowered lateral spikelets [13]. In *Eurychorda*, by contrast, both sexes retain true spikelets aggregated into a paniculate synflorescence. This contrast reveals that sexual dimorphism in Restionaceae can operate at the level of the basic flowering unit rather than being restricted to floral organ number, fitting broader patterns in dioecious plants where dimorphism frequently involves inflorescence structure, condensation and packing of reproductive modules [22,23,24]. Importantly, the dimorphism does not require wholesale reorganization of the synflorescence: the same underlying ramification pattern is retained, with divergence arising from local changes in the structure, number and repetition of terminal modules. This kind of “re-writing” on a conserved architectural scaffold is consistent with comparative analyses showing that small shifts in branching rank, paraclade length, and meristem fate can generate major diversity in inflorescence form without altering overall synflorescence topology [17]. At present, however, the processes generating these differences remain unresolved. Scenarios deserving further study include a modest shift in branching rank and timing of lateral initiation within the female flowering units. Progress will require an integrated ontogenetic series, detailed analysis of reproductive-shoot architecture, and comparative tests on robust phylogenies to assess character polarity and correlated evolution.

Because the term *spikelet* has been used inconsistently across Poales [13], we adopt a strict definition for Restionaceae: a racemose inflorescence with an axis bearing lateral, sessile flowers [19,25]. Under this usage, both male and female units of *Eurychorda* are true spikelets, whereas the female unit of *L. denmarkicus* constitutes a compound spike—i.e., a secondary axis bearing a series of reduced, one-flower lateral spikelets that together mimic a single unit. This reframing clarifies homology by avoiding category collapse in which compound structures are mislabeled as spikelets *sensu stricto*. It also aligns terminology with ontogeny: our developmental observations indicate that the compound female spike in *L. denmarkicus* arises through segmentation of the female module, with lateral initiation shifted upward in rank. Treating these alternatives as distinct flowering units (true spikelet versus compound spike) enables meaningful comparison of building rules across taxa and sexes. It also cautions against transferring grass-centric spikelet concepts [9] uncritically to Restionaceae, where reduction and modular repetition are pervasive and can produce deceptive “pseudo-spikelets”.

Given the basal position of *Eurychorda* within Leptocarpoideae [14], the occurrence of true spikelets in both sexes may be the most parsimonious ancestral condition for the subfamily, with the compound female spike in *L. denmarkicus* [13,26] interpreted as a derived condition. This polarity implies the female-biased modification evolved on a conserved scaffold, perhaps, via shifts in branching hierarchy [27,28]. Such changes can plausibly originate through small adjustments in growth rates or thresholds governing lateral initiation. Mapping inflorescence traits onto backbone phylogenies [14] will allow formal tests of directionality and help distinguish repeated derivations from a single origin of the compound condition [29,30].

**Reorganizations in androecium and gynoecium** are **associated with sexual dimorphism.** Restionaceae are predominantly dioecious, a condition frequently linked to wind pollination [14,25]. However, some exceptions occur: several species are monoecious, bearing bisexual or unisexual flowers [14]. The most conspicuous monoecious lineage is the centrolepids (now Restionaceae: Centrolepidoideae), in which a single stamen develops alongside multiple carpels arranged in a row and covered by phyllomes [31]. Ontogenetic data indicate that these complexes represent highly derived bisexual flowers formed by reduction of the androecium and oligomerization of the gynoecium [32,33], countering earlier interpretations that treated them as condensed, mixed inflorescences comprising a male flower plus many monocarpellate female flowers [34,35].

Beyond Centrolepidoideae, many monothecal restionaceous taxa bear functionally unisexual flowers [12,36,37], although in a few species both sexes occur on the same plant, e.g., *Lepyrodia hermaphrodita*, *L. fortunata* [14]. Developmental series across the family show a common pattern: both androecial and gynoecial primordia typically initiate [12,36,37], after which one set arrests at varying checkpoints, yielding a continuum from subtle vestiges (staminodes and pistillodes) to complete functional unisexuality. Staminodes range from minute undifferentiated bulges (e.g., *Hopkinsia*) to unvascularized filiform elements (*Anarthria*) [12] and, in some cases, nearly complete vascularized anther-plus-filament structures. In *Eurychorda*, arrest chiefly suppresses anther development while the filament elongates markedly; the gynoecium may cease late, after carpel differentiation and ovule initiation, with carpel margins remaining unfused. In such a pistillode, even the vascular system is retained. Occasionally, a tiny undifferentiated pyramidal protrusion is present at the center of male flowers [11]; interpreting structures of this kind on developmental and anatomical evidence alone is challenging [38]. Given the diversity of staminodes [12,36,37] and the presence of a pistillode [14] in some flowers, the evolution of reduced organs in Restionaceae and the developmental pathways [36,37] underlying their formation [1,39,40] deserve further study, including potential reversals between uni- and bisexual flowers [41,42].

Taken together, these observations support a shared ontogenetic program in which timing (heterochrony) and degree (allometry) of arrest generate the observed diversity of reductions [1,39,40,43,44,45]. Recognizing reduction and arrest as primary drivers helps reconcile conflicting treatments of “unisexuality,” explains the wide range of vestigial states generally found [1,39], and frames testable hypotheses about their regulation [40,45]. Progress will require integrating dense ontogenetic series, quantitative scoring of arrest checkpoints [1] (e.g., stage of anther differentiation, carpel-margin fusion), and comparative analyses on robust phylogenies to test for repeated origins, polarity [29,30,39], and potential reversal between uni- and bisexual conditions [41,42].

**Wind-pollination mechanics.** Wind pollination pervades the family [46,47] and covaries with familiar traits—dioecy, perianth reduction, abundant pollen, few ovules, and the development of branched stigmas [23]—but our comparison shows that the mechanics of pollen liberation can be achieved via alternative morphological routes. In *Eurychorda*, anthers are exserted on long, flexible filaments from an opening perianth at anthesis [48]; the resulting pendulous anthers are highly efficient pollen shakers under low-to-moderate turbulence. Biomechanical studies show that in wind-pollinated angiosperms, long, slender, flexible filaments resonate in turbulent air, releasing pollen in bursts; this mechanism is effective even at low average wind speeds [49,50,51]. In *L. denmarkicus*, by contrast, filaments are very short and anthers remain within the perianth, but male spikelets sit on long, flexible peduncles that invert the spikelet upside down [13,52,53] and promote trembling, thereby ventilating the perianth chamber and aiding pollen escape. These two solutions—filament elongation versus spikelet peduncle flexibility—represent reciprocal mechanical strategies that likely reflect differences in exposure, boundary-layer conditions, and construction cost. The observations that several other genera of Restionaceae (e.g., *Apodasmia*, *Chaetanthus*, *Hypolaena*, *Leptocarpus*) also bear pendulous or mobile male spikelets [8] suggest repeated exploration of this functional axis within the family. By treating pollination as a biomechanical problem, we can relate specific structural parameters (filament length, perianth aperture, spikelet peduncle stiffness) to aerodynamic performance and thereby generate comparative metrics suitable for hypothesis testing [49].

Taken together, our observations suggest several testable expectations. If the compound female spike in *L. denmarkicus* [13] reflects modular segmentation, we should observe upward shifts in branching rank and altered timing of lateral initiation [27,28] within female units relative to males and to *Eurychorda*. Treating *true spikelet* and *compound spike* as distinct states should improve the fit of comparative models that allow different transition rates between these units. If reductions in the androecium and gynoecium primarily reflect heterochronic arrest, then the stage of arrest—such as the onset of anther-wall differentiation, the extent of filament elongation, and the timing of carpel-margin fusion—should covary across taxa and align with the observed spectrum of staminodes and pistillodes [39,40], including possible reversals toward more complete bisexual expression [1]. Because wind pollination can be realized by alternative structural solutions, we further expect reciprocal compensation among filament length, degree of perianth opening, and spikelet-peduncle flexibility while yielding comparable pollen release under standardized airflow [28,49,50]. Finally, if *Eurychorda* approximates the ancestral condition for Leptocarpoideae [14], character mapping on backbone phylogenies [29,30] should favor true spikelets in both sexes as the most parsimonious state, with independent derivations of compound female spikes.

## 4. Materials and Methods

*Eurychorda complanata* (R.Br.) B.G.Briggs & L.A.S.Johnson [48] is common in Tasmania but distributed across south-eastern Australia (Victoria, New South Wales, Queensland, South Australia). It was studied using material collected in New South Wales, Australia, in October 2014 (voucher: *B.G.Briggs 10166*, *10167*; voucher deposited at NSW). Material was fixed and stored in 70% ethanol until examination. Inflorescence architecture and external floral morphology were examined under an Olympus SZX7 stereomicroscope (Olympus, Tokyo, Japan). The morphology and development of reproductive organs were studied with a CamScan S-2 scanning electron microscope (Cambridge Instruments, London, UK). Prior to imaging, material was dehydrated through an organic-solvent series: 96% ethanol (2 × 30 min), 96% ethanol:100% acetone (1:1, 30 min), and 100% acetone (3 × 30 min). Samples were then critical-point dried (Hitachi HCP-2: Hitachi, Tokyo, Japan), mounted onto specimen stubs using Carbon Conductive Tabs (Ted Pella, Inc., Redding, CA, USA) and sputter-coated with gold-palladium (Giko IB-3: JEOL, Tokyo, Japan). SEM work was conducted at the Laboratory of Electron Microscopy, Faculty of Biology, Lomonosov Moscow State University.

The sections were prepared using Technovit 7100 resin (Kulzer Technik, Wehrheim, Germany) [54]. Embedded material was sectioned at 5 µm with an HM 340E automated rotary microtome (Thermo Scientific, Waltham, MA, USA), stained with 0.5% toluidine blue in 1% aqueous acetic acid, and mounted in BioMount (Bio-Optica, Milan, Italy). Sections were studied with an Olympus CX31 light microscope (Olympus, Tokyo, Japan). Selected sections were photographed using an Olympus SC50 camera (Olympus, Tokyo, Japan) with Olympus cellSens Entry 2.1 software (Olympus, Tokyo, Japan).

SEM micrographs, LM images, and scientific drawings were processed and assembled in Adobe Illustrator and Adobe Photoshop (Adobe Inc., San Jose, CA, USA). Terminology follows Fomichev et al. [13].

## Data Availability

The original contributions presented in this study are included in the article. Further inquiries can be directed to the corresponding author.
